# Supplementary material for: SubClonal Hierarchy Inference from Somatic Mutations: Automatic Reconstruction of Cancer Evolutionary Trees from Multi-region Next Generation Sequencing
Source: PLoS Comput Biol. 2015 Oct 5;11(10):e1004416. doi: 10.1371/journal.pcbi.1004416 (PMC4593588; doi:10.1371/journal.pcbi.1004416)
Supplement: S1 Text — (PDF) [file pcbi.1004416.s001.pdf]

SubClonal Hierarchy Inference from Somatic Mutations: automatic reconstruction of cancer evolutionary trees from multi-region next generation sequencing

Noushin Niknafs[1], Violeta Beleva-Guthrie[1], Daniel Q. Naiman[2], Rachel Karchin[1,3,*]

1 Department of Biomedical Engineering and Institute for Computational Medicine, Johns Hopkins University, Baltimore, MD, USA
2 Department of Applied Math and Statistics, Johns Hopkins University, Baltimore, MD, USA
3 Department of Oncology, Johns Hopkins Medical Institutions, Baltimore, MD, USA
* E-mail: karchin@jhu.edu

Supplementary Methods

Naive mutation cellularity estimate

In diploid regions, a maximum likelihood estimate based on the observed variant allele fraction of a mutation can be used to infer its cellularity. We use the following simple derivation to estimate the cellularity and its standard error from reference and variant read counts for a mutation i in a diploid region of the genome. We further assume that the genotype of normal cells and non-variant tumor cells (tumor cells not carrying the mutation) is AA where A is the reference allele, and there is no loss of heterozygosity. Under these conditions, the probability of sampling a variant allele from tumor sample s with purity α^s, for mutation i with cellularity C_i^s is

$$V_{exp} = \frac{\alpha^s \cdot C_i^s}{2} \tag{S1}$$

where V_{exp} is the expected variant allele fraction. By the binomial read count assumption, $\frac{r_B}{r_T}$ is a single sample unbiased estimator of V_{exp}; here r_B and r_T represent the observed variant and total read count of a mutation, respectively.

$$r_B \sim binomial(r_T, V_{exp}) \tag{S2}$$

$$V_{obs} = \frac{r_B}{r_T} \tag{S3}$$

Therefore, mutation cellularity can be estimated as

$$\hat{C}_i^s = \frac{2\ V_{obs}}{\alpha^s} \tag{S4}$$

Finally, the estimated variance of the above estimator is

$$\sigma_i^{s\ 2} = \frac{4}{\alpha^{s\ 2}} \sigma^2_{V_{obs}} \tag{S5}$$

$$= \frac{4}{\alpha^{s\ 2}} \frac{V_{exp}\ (1 - V_{exp})}{r_T} \tag{S6}$$

$$\sigma_i^{s\ 2} \approx \frac{4}{\alpha^{s\ 2}} \frac{V_{obs}\ (1 - V_{obs})}{r_T} \tag{S7}$$

Equation S7 shows that the variance of the cellularity estimates decreases as purity and coverage increase.

Note that a simple modification allows us to extend this approach to regions with copy number = 1. For mutations in these regions, the cellularity is

$$\hat{C}_i^s = \frac{(2 - \alpha^s)\ V_{obs}}{\alpha^s} \tag{S8}$$

The estimated variance is

$$\sigma_i^{s\ 2} \approx \frac{(2 - \alpha^s)^2}{\alpha^{s\ 2}} \frac{V_{obs}\ (1 - V_{obs})}{r_T} \tag{S9}$$

Naive cellularity estimation is useful for mutations located in diploid or hemizygous regions of cancer genomes but is of limited utility in genomes with widespread aneuploidy.

In patients with largely diploid genomes, it is possible to follow the approach above and in each tumor sample, treat the cellularity of mutations outside diploid or hemizygous regions as missing values. In the next step, for each pair of mutations/mutation clusters, the SCHISM hypothesis test can be performed—although with reduced power—by excluding any samples in which any member of the pair had missing cellularity values. On the other hand in patients with largely aneuploid genomes, more sophisticated approaches to estimate cellularity values are recommended [1, 2]. These approaches can handle variable ploidy states and by providing cellularity estimates for mutations in a larger subset of samples can help increase the power of the hypothesis test.

Cluster cellularity estimation

By definition, all mutations in a cluster are assumed to have the same cellularity in each sample. If the cellularity of individual mutations is available, the cellularity of a cluster is estimated to be the mean cellularity of its members

$$CC[I, s] = \frac{\sum\limits_{i \in I} C_i^s}{|M(I)|}. \tag{S10}$$

Otherwise cluster cellularity values from other sources can be used. The cellularity of each cluster across multiple samples is represented as a a matrix CC whose elements report the cellularity of each cluster I, for each sample s.

Topology Similarity Measure

We apply the *Jaccard Index* to quantify the degree of similarity between two tree topologies—sharing the same mutation clusters—as follows. Let S_u be the set of all mutation cluster pairs (I, J) where mutation cluster I is in the same lineage as and precedes mutation cluster J in tree u. Then the similarity of two subclonal phylogeny trees u and v can be measured as:

$$JI = \frac{|S_u \cap S_v|}{|S_u \cup S_v|} \tag{S11}$$

By construction, Jaccard index varies in $[0, 1]$. A value of 1 for the Jaccard Index indicates equality of sets S_u and S_v and thus identical topologies for trees u and v.

Simulations

Generating mutation cellularities

In each simulated sample s, a breadth-first-search (BFS) of a subclone tree begins at the incident edge downstream of the root node. The mutation cluster corresponding to this edge has cellularity of 1, and it

represents clonal mutations occurring in the most recent common progenitor cell of all the patient's tumor cells. For subsequent edges, cellularity values are distributed with a modified version of the tree-structured stick-breaking process model [3, 4].

For each tree topology *instance* at each node count level, ten sets of mutation cluster cellularity values are generated, representing 10 samples from an individual. Each tree node then represents a unique set of cells or subclones harboring mutations, which have accumulated along the path from the root to that node. As in (Methods Equation 31), $p(n)$ is the mutation cluster associated with the edge immediately upstream of node n, and $D(n)$ is the set of mutation clusters associated with its immediate downstream edges. Letting $n = 0$ correspond to the node immediately downstream of the root, the edge corresponding to clonal mutations is assigned cellularity $C^s_{p(0)} = 1$. For each subsequent node n, the fraction of tumor cells that harbor $p(n)$ but none of the mutation clusters downstream of n is $C^s_{p(n)}\omega(n)^s$ where

$$\omega(n)^s \sim B\left(\alpha_b = 1, \beta_b = \alpha_0\ \lambda_0^{(d_n-1)}\right) \tag{S12}$$

and $\alpha_0 = 5$, $\lambda_0 = 0.5$ and d_n is the depth of node n in the rooted tree. Then $C^s_{p(n)}(1 - \omega(n)^s)$ is the fraction of cancer cells harboring at least one mutation cluster in $D(n)$ in addition to $p(n)$. These are cells that diverge from their parental population at node n. Therefore,

$$\sum_{q \in D(n)} C^s_q = C^s_{p(n)}(1 - \omega(n)^s) \tag{S13}$$

Letting V be a vector of size $|D(n)|$, and

$$V \sim Dir\left(\alpha_{dir} = 1\right) \tag{S14}$$

then the cellularity of each downstream mutation cluster is

$$[C^s_q, q \in D(n)] = V C^s_{p(n)}(1 - \omega(n)^s) \tag{S15}$$

To capture variability among individuals, this procedure is replicated ten times for each tree *instance.*

Mutation Variant Allele Fractions

To obtain read counts that are consistent with the simulated cellularity values in each sample s, variant and reference read count values are generated for each mutation in mutation cluster $p(n)$ associated with the edge immediately upstream of node n, given simulated cellularity value $C^s_{p(n)}$, as follows. We make the simplifying assumption that mutations considered in this simulation experiment are located in diploid regions of tumor genomes in all samples. This assumption enables application of the naive cellularity estimator and allows focusing on performance of the other modules in SCHISM pipeline. Following the conditions above, the expected variant allele frequency for mutations in $p(n)$ is

$$V^{p(n)}_{exp} = \frac{\alpha^s C^s_{p(n)}}{2} \tag{S16}$$

Since read counts may be overdispersed, a noisy expected variant allele frequency for each mutation $i \in p(n)$ is generated as

$$S \sim \Gamma\left(k = 10000, \theta = 1\right) \tag{S17}$$

$$V^i_{exp} = B\left(S\ V^{p(n)}_{exp}, S\ \left(1 - V^{p(n)}_{exp}\right)\right) \tag{S18}$$

where S is a global precision parameter for each simulated individual. Given coverage r^i_T for variant i, variant read count is

$$r^i_B \sim binom\left(r^i_T, V^i_{exp}\right) \tag{S19}$$

Supplementary Results

Genetic algorithm evaluation with large subclone count

To explore the utility of the genetic algorithm presented in this work when the number of nodes in a subclonal phylogeny is large, we designed a simulation in which 7 tumor samples were available from a patient and 15 subclones were present. In the simulations, the true subclonal phylogeny tree and the cellularity of each mutation cluster in each sample is known. Two patients were simulated. For one patient, the topology of the true tree was predominantly branched, and for the other the topology was predominantly linear (Fig. S5A, Fig. S6A). Mutation cluster cellularities for each patient are shown in (Fig. S5B, Fig. S6B). The hypothesis test was applied to each pair of mutation clusters, yielding CPOV matrices (Fig. S5C, Fig. S6C). For each simulated patient, we performed 10 runs of the GA, where each run consisted of 50 generations with 1000 trees per generation. To assess how many generations were required to identify the true tree, we monitored the progress of the GA by evaluating the topologies of all trees in the population reported to have maximum fitness after each generation. Evaluation was done by computing a Jaccard index of each of these trees with respect to the true tree (Text S1, Topology Similarity Measure). The maximum Jaccard Index after each generation is shown in (Fig. S5D, Fig. S6D).

Fig. S5 shows results for one of the simulated patients, in which the true tree topology is primarily branched. For this patient, 8 of the 10 GA runs identified the true tree at 50 generations (Fig. S5D). Fig. S5E shows the progress of one GA run that was randomly selected from these 8 runs. For each generation, the Jaccard Index for all of the maximum fitness trees is shown, rather than only for the tree with maximum Jaccard Index. At the 50th generation, the GA identified nine trees with maximum fitness and one of them was the true tree (Fig. S5E). A consensus tree of these nine trees is shown in Fig. S5F.

Fig. S6 show results for the second simulated patient, in which the true tree topology is primarily linear. For this patient, 1 of the 10 GA runs identified the true tree at 50 generations (Fig. S6D). Fig. S6E shows the progress of this GA run. At the 50th generation, the GA identified 17 trees with maximum fitness and one of them was the true tree (Fig. S6E). A consensus tree of these 17 trees is shown in Fig. S6F.

These results suggest that the GA is able to identify the true subclonal phylogeny, even in cases where the number of subclones is as high as 15, and it is reasonable to infer that it will even succeed for larger numbers of subclones, in some but not all cases. Importantly, the effectiveness of the GA may depend on details of the patient's cancer evolutionary history and the biopsy samples.

Limitations of topology cost or mass cost alone in identifying the true tree

Our fitness function depends on both a topology cost (TC), represented in the CPOV matrix, and a mass cost (MC). The effectiveness of the TC by itself in narrowing down candidate tree topologies and identifying the true tree depends on the topology of that true tree. For completely branched topologies, the TC alone may be sufficient in identifying the true tree, if the CPOV matrix has power of 1.0 and Type 1 error of 0. In the case of trees that include linear topologies, the TC alone will not be sufficient. Fig. S1 shows two scenarios that illustrate this point. For the 5-node branched tree (Fig. S1A) the CPOV matrix alone can identify the true tree. For the 5-node linear tree (Fig. S1B), six candidate trees (Fig. S1C) are equally likely to be the true tree if only the CPOV matrix is considered.

Fig. S2 shows an example where MC alone is not sufficient to identify the true tree, a five-node tree representing a moderately branched evolutionary pattern (Fig. S2A). Fig. S2B and Fig. S2C show cellularity values for two simulated samples and the CPOV matrix. Five candidate tree topologies (Fig. S2D) are depicted. Topogies D1, D2 and D3 have minimum TC; D4 and D5 have minimum MC. Using both TC and MC uniquely identifies the true tree topology (D1).

Sensitivity of genetic algorithm to f_c parameter

The fitness function in our genetic algorithm is a decaying exponential function of tree cost. The parameter f_c is a coefficient of the cost and controls the rate at which changes in cost yield changes in fitness. We performed a sensitivity analysis to identify a good default value f_c on simulated data. Two scenarios were considered: 8-node tree with three samples and 15-node tree with seven samples. We ran 20 independent runs of the GA for each tree, using 6 unique values of f_c. A run for the 8-node tree spanned 20 generations, and a run for the 15-node tree spanned 50 generations, to enable good sampling of tree topology space. Each generation consisted of 1000 trees. The value $f_c = 5$ produces the best results, when both scenarios are considered. For the 8-node tree, 19 out of 20 runs identified the true tree and for the 15-node tree, 17 out of 20 runs identified the true tree (Fig. S7).

Detail of cost function for multi-sample sequencing studies

Table S2 shows the performance of SCHISM on data from the three multi-sample sequencing studies (Results) when only the topology cost (TC) or mass cost (MC) is used in the genetic algorithm. Addition of MC substantially reduces the number of maximum fitness trees identified. In this set of samples, MC alone has equivalent performance to TC+MC. As shown in (Fig. S1, Fig. S2), inclusion of both cost terms can handle scenarios where only one is insufficient.

Comparison of SCHISM, SubcloneSeeker and TrAp output on patient AML1

Subclone Seeker and TrAp have similar inputs to SCHISM but the modeling task and outputs are different. SCHISM models a single, unified tree across multiple samples. Subclone Seeker and TrAp model trees for each individual sample and do not provide a unified tree. To illustrate these differences, we have compared the output of SCHISM to SubcloneSeeker and TrAp on eight two-sample cases (primary and relapse) from acute myeloid leukemia (AML) patients [5] and one two-sample case (primary and metastasis) from a murine small cell lung cancer (mSCLC) [6]. (Subclone Seeker can handle only up to two samples). Variant allele fractions and mutation cluster assignments from [5] were used to estimate cluster cellularities and these were input to all three methods.

Fig. S8 shows detailed results for the AML patient AML1.

SCHISM results are a unified tree for both samples in patient AML1 (Fig. S8A).

SubcloneSeeker models the evolution of each individual sample, then identifies compatible pairs of sample trees (Fig. S8B). Six trees are reported for the AML1 primary sample (Primary Tree 1, Primary Tree 6, Primary Tree 11, Primary Tree 16, Primary Tree 21, Primary Tree 26). One tree is reported for the secondary sample (Secondary Tree 1). Primary Tree 11 is reported as compatible with Secondary Tree 1 (marked with asterisks in Fig. S8B). As shown, each tree node is labeled with n followed by a number. The labels are not related to user-provided mutation cluster IDs. It is unclear how Primary Tree 11 and Secondary Tree 1 should be merged, given that the node numbers are disjoint between the two trees, or whether the two trees are compatible with the SCHISM unified tree.

TrAp also models the evolution of each individual sample, requiring compatibility across all samples, then outputs individual sample trees (Fig. S8C). The tree on the left represents the AML1 primary sample and the tree on the right represents the relapse sample. The TrAp trees are compatible with the SCHISM tree, which matches the manually curated tree published by Ding et al. However, the TrAp trees collapse two of the mutation clusters in the primary sample and three in the relapse sample, which is likely due to over-merging of mutation clusters.

SubcloneSeeker and TrAp appear to over-merge mutation clusters in three and two of the seven remaining AML patients, respectively. For mSCLC case mSCLC984, SubcloneSeeker does not generate any compatible pairs for the primary and secondary samples, and TrAp does not generate any trees compatible with unified trees identified by SCHISM, or the manually curated tree in [6].

References

1. Roth A, Khattra J, Yap D, Wan A, Laks E, Biele J, et al. PyClone: statistical inference of clonal population structure in cancer. Nature Methods. 2014;.

2. Miller CA, White BS, Dees ND, Griffith M, Welch JS, Griffith OL, et al. SciClone: Inferring clonal architecture and tracking the spatial and temporal patterns of tumor evolution. PLoS Computational Biology. 2014;10(8):e1003665.

3. Ghahramani Z, Jordan MI, Adams RP. Tree-structured stick breaking for hierarchical data. In: Advances in Neural Information Processing Systems; 2010. p. 19–27.

4. Jiao W, Vembu S, Deshwar AG, Stein L, Morris Q. Inferring clonal evolution of tumors from single nucleotide somatic mutations. BMC Bioinformatics. 2014;15(1):35.

5. Ding L, Ley TJ, Larson DE, Miller CA, Koboldt DC, Welch JS, et al. Clonal evolution in relapsed acute myeloid leukaemia revealed by whole-genome sequencing. Nature. 2012;481(7382):506–510.

6. McFadden DG, Papagiannakopoulos T, Taylor-Weiner A, Stewart C, Carter SL, Cibulskis K, et al. Genetic and clonal dissection of murine small cell lung carcinoma progression by genome sequencing. Cell. 2014;156(6):1298–1311.
