# Supplementary figures and images for: SubClonal Hierarchy Inference from Somatic Mutations: Automatic Reconstruction of Cancer Evolutionary Trees from Multi-region Next Generation Sequencing
Source: PLoS Comput Biol. 2015 Oct 5;11(10):e1004416. doi: 10.1371/journal.pcbi.1004416 (PMC4593588; doi:10.1371/journal.pcbi.1004416)

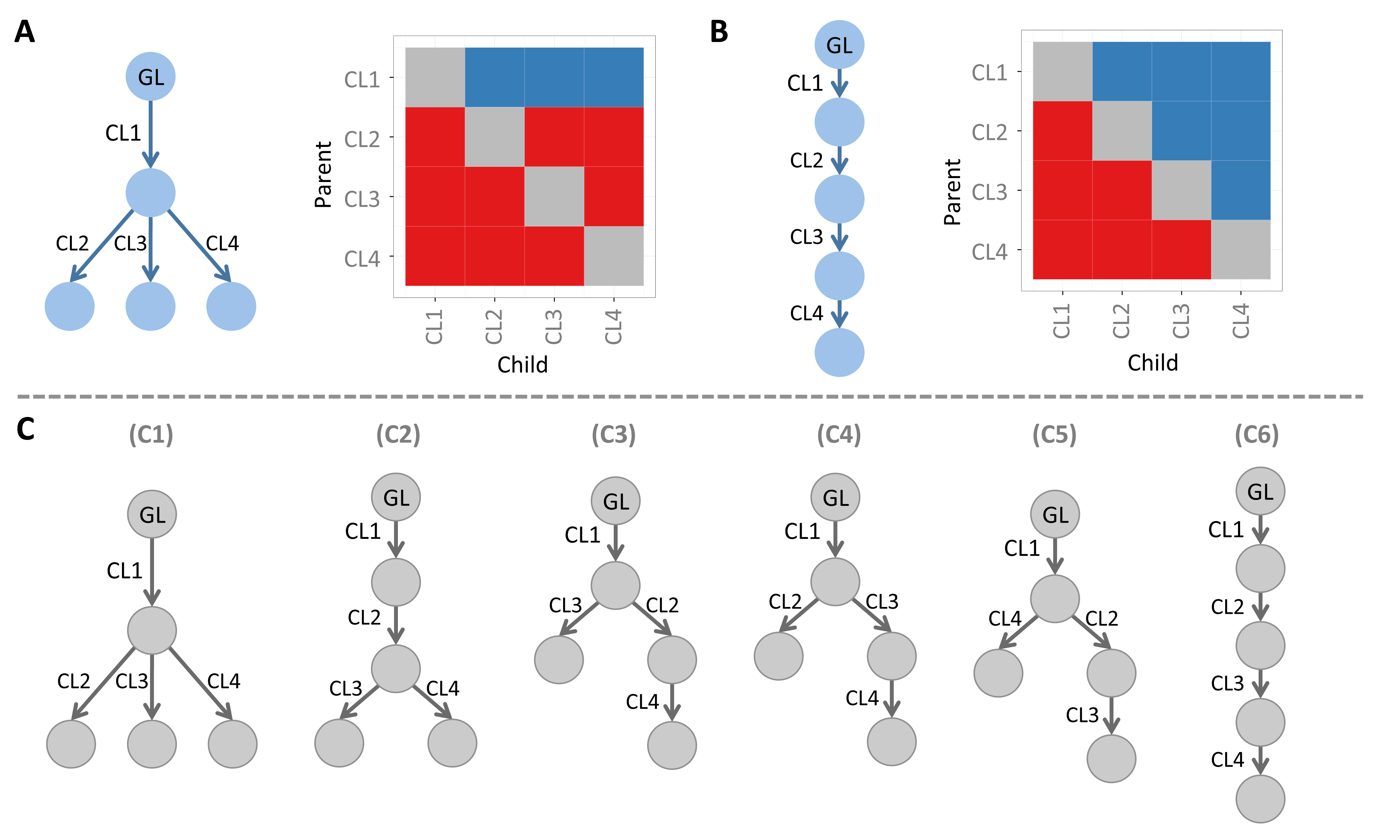

Supplement: S1 Fig — Our fitness function considers both a topology cost and a mass cost. If only topology cost is used, in the case of a completely branched tree (A) and a CPOV matrix with power of 1.0 and Type 1 error of 0, the mass cost is not necessary to narrow down candidate tree topologies. However, if the tree contains linear topologies (B), the topology cost is not sufficient to identify the true tree. A and B. simple 5-node trees representing branched (A) and linear (B) evolutionary patterns. CPOV matrices under the assumption of power 1.0 and Type error 0 appear to the right of each tree. C. Candidate tree topologies. C1 depicts the only tree topology compatible with the CPOV matrix of the branched evolutionary pattern (A); while C1–6 are all compatible with the CPOV matrix of the linear evolutionary pattern (B), showing that in this case the topology cost alone is not sufficient. (TIF) [file pcbi.1004416.s002.tif]

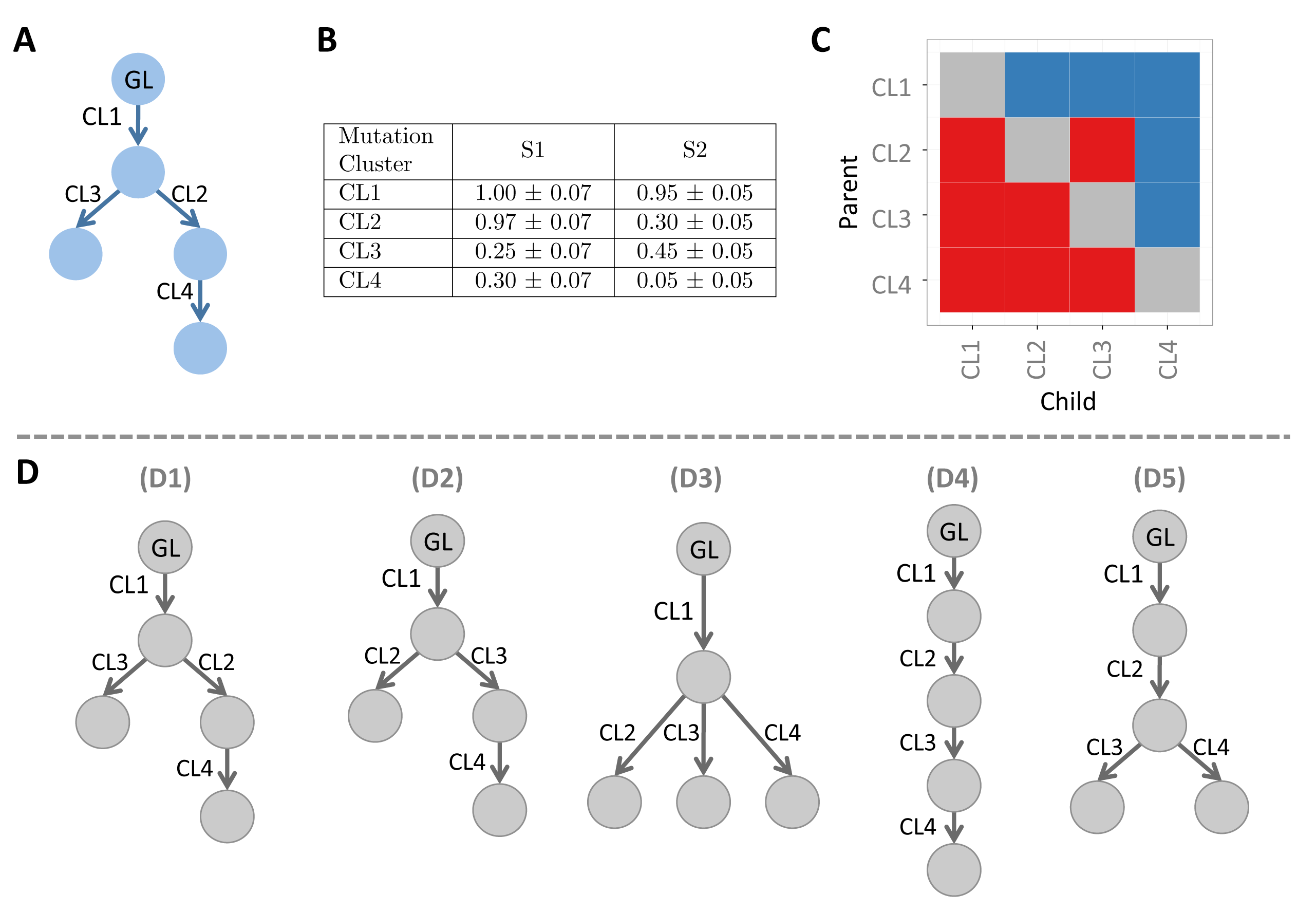

Supplement: S2 Fig — Our fitness function considers both a topology cost and a mass cost. Mass cost alone is not sufficient to identify the true tree topology for the example cluster cellularity input in (B). The combination of the two cost terms outperforms each term alone in this example. A simple 5-node tree representing a moderately branched evolutionary pattern. B. Input cellularity values in two simulated samples. C. CPOV matrix. D. Candidate tree topologies. D1–3 depict topologies with minimum topology cost, and D4–5 depict those with minimum mass cost. Using both topology and mass cost uniquely identifies the true tree topology (D1). (TIFF) [file pcbi.1004416.s003.tiff]

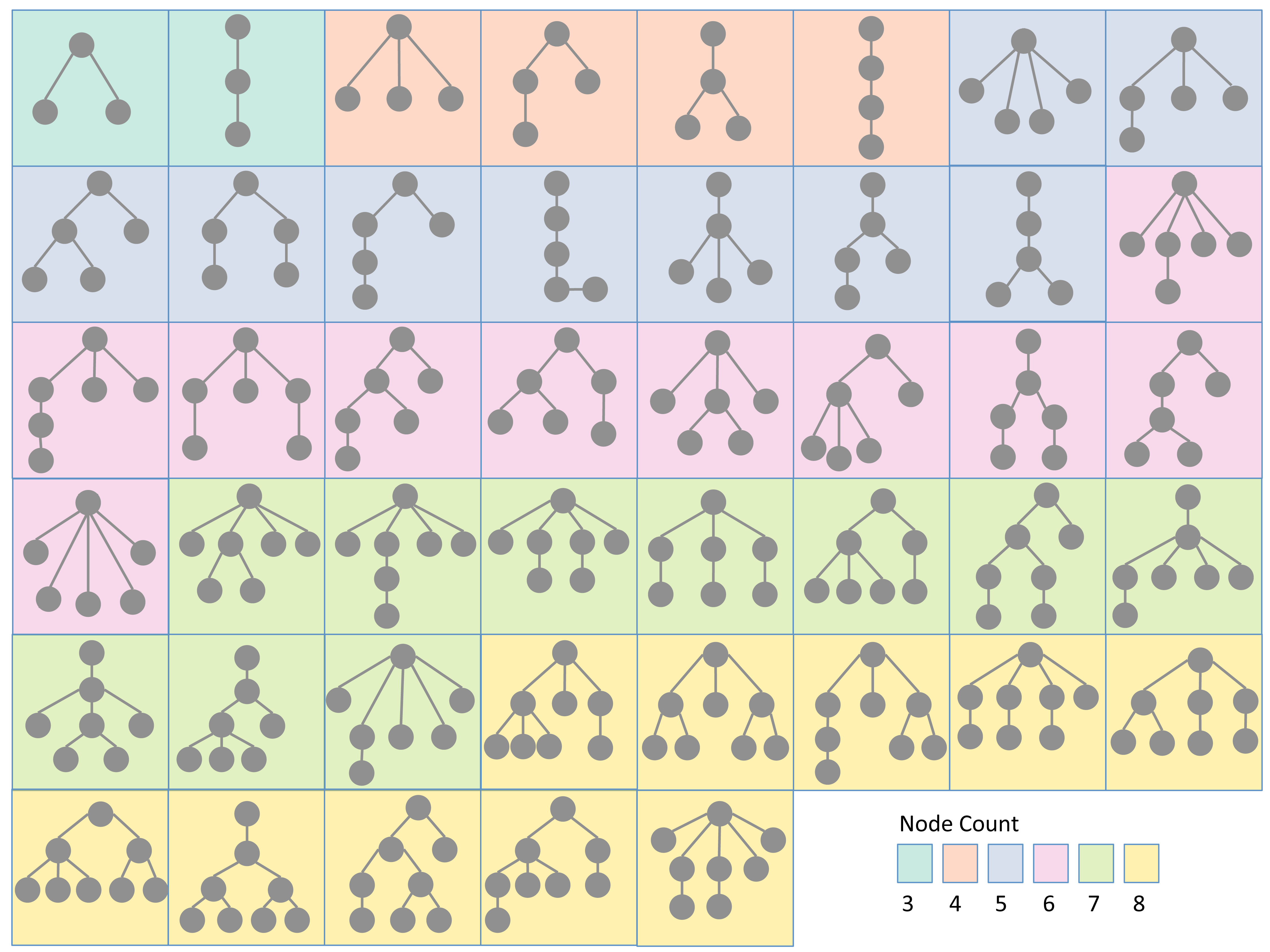

Supplement: S3 Fig — For trees with 3–5 nodes, the exhaustive set of possible topologies were used. Otherwise, we manually selected ten topologies. Each box depicts a tree instance. (TIF) [file pcbi.1004416.s004.tif]

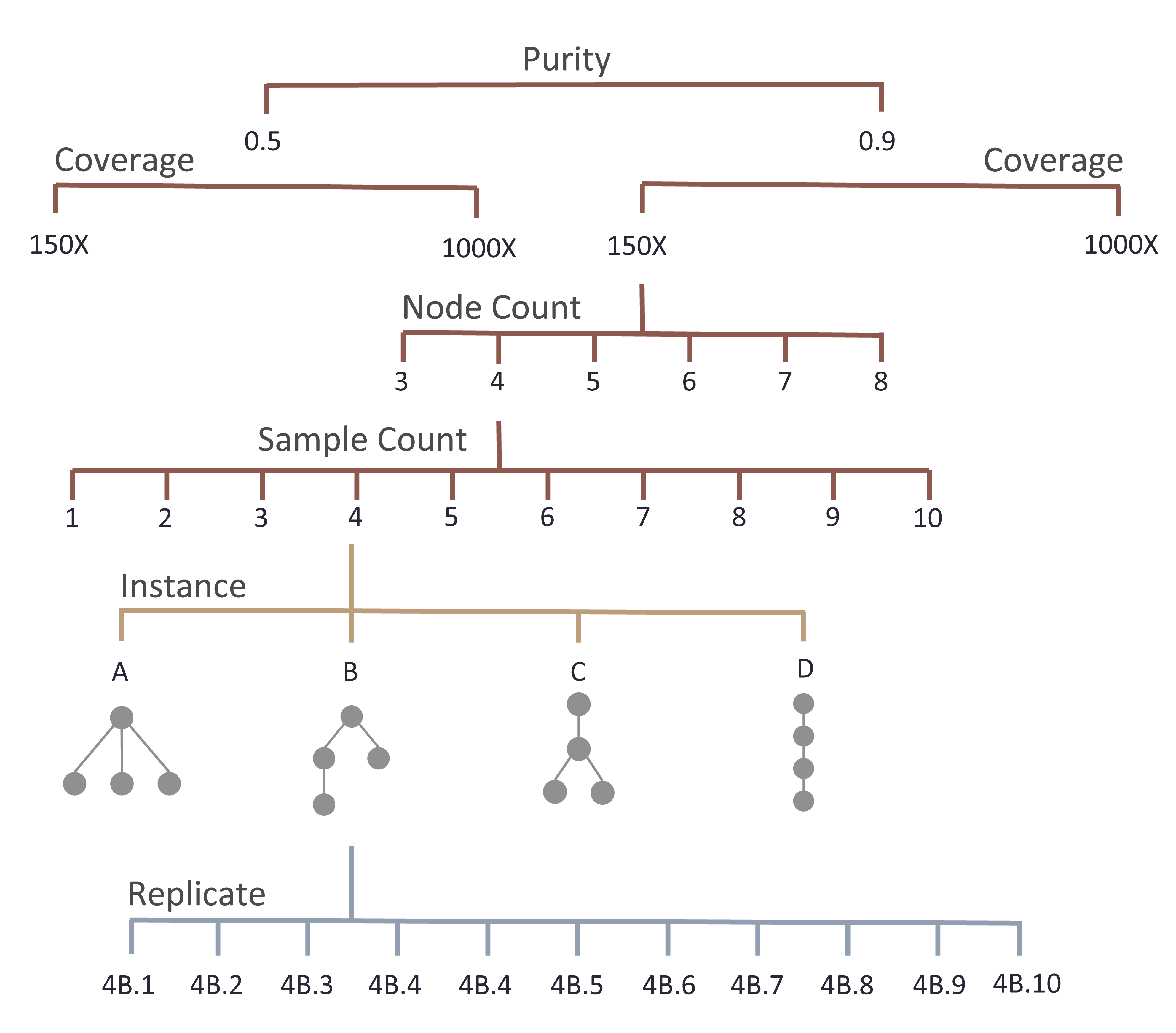

Supplement: S4 Fig — The example shows purity of 0.9, coverage of 150X, node count of 4, sample count of 4, and a selected tree instance. (TIF) [file pcbi.1004416.s005.tif]

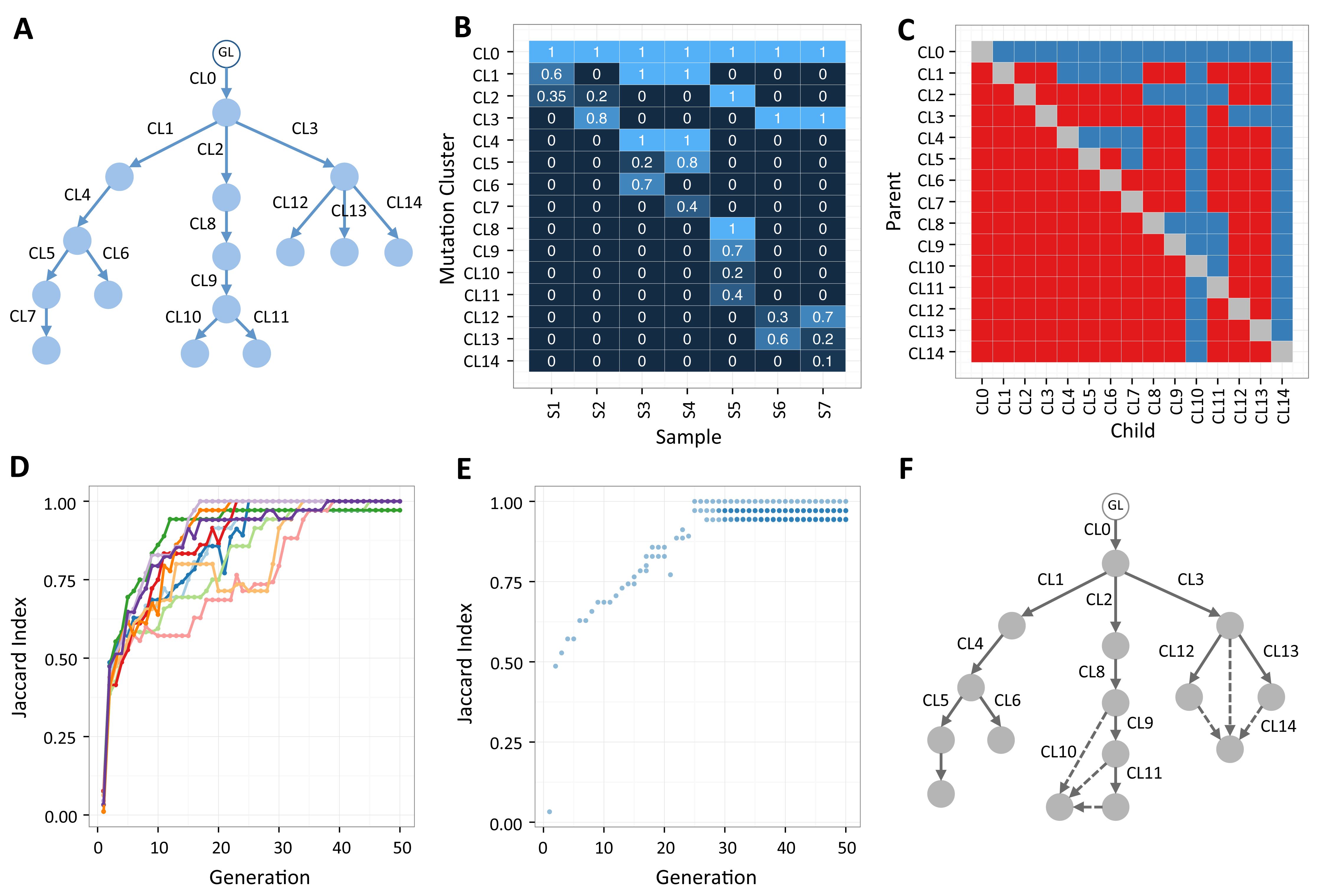

Supplement: S5 Fig — A. a phylogenetic tree summarizing clonal evolution in a simulated patient B. Cellularity values of fifteen mutation clusters (CL0–14) in seven simulated biopsies S1–7 C. CPOV matrix depicting the hypothesis test outcomes. Each red square represents a pair of mutation clusters (I,J) for which the null hypothesis that I could be the parent of J was rejected. Each blue square represents a pair for which the null hypothesis could not be rejected. D. The Jaccard index (S1 Text, Topology Similarity Measure) of the true tree (A) and the maximum fitness tree(s) in population at the end of each GA generation. In cases where more than a single highest maximum fitness tree was present, the maximum of the Jaccard indices is plotted. Each color trace represent one of ten independent GA runs performed on inputs in (B, C). E. The Jaccard index of the true tree and the maximum fitness trees at the end of each generation for a sample GA run. Each transparent circle represents a single maximum fitness tree. This GA run identified 9 maximum fitness tree topologies, one of which was the true tree (A). F. The consensus topology of the nine trees identified in (E). These trees shared the majority of their lineage relationships and only disagreed in parental lineage of mutation clusters CL10 and CL14. (TIF) [file pcbi.1004416.s006.tif]

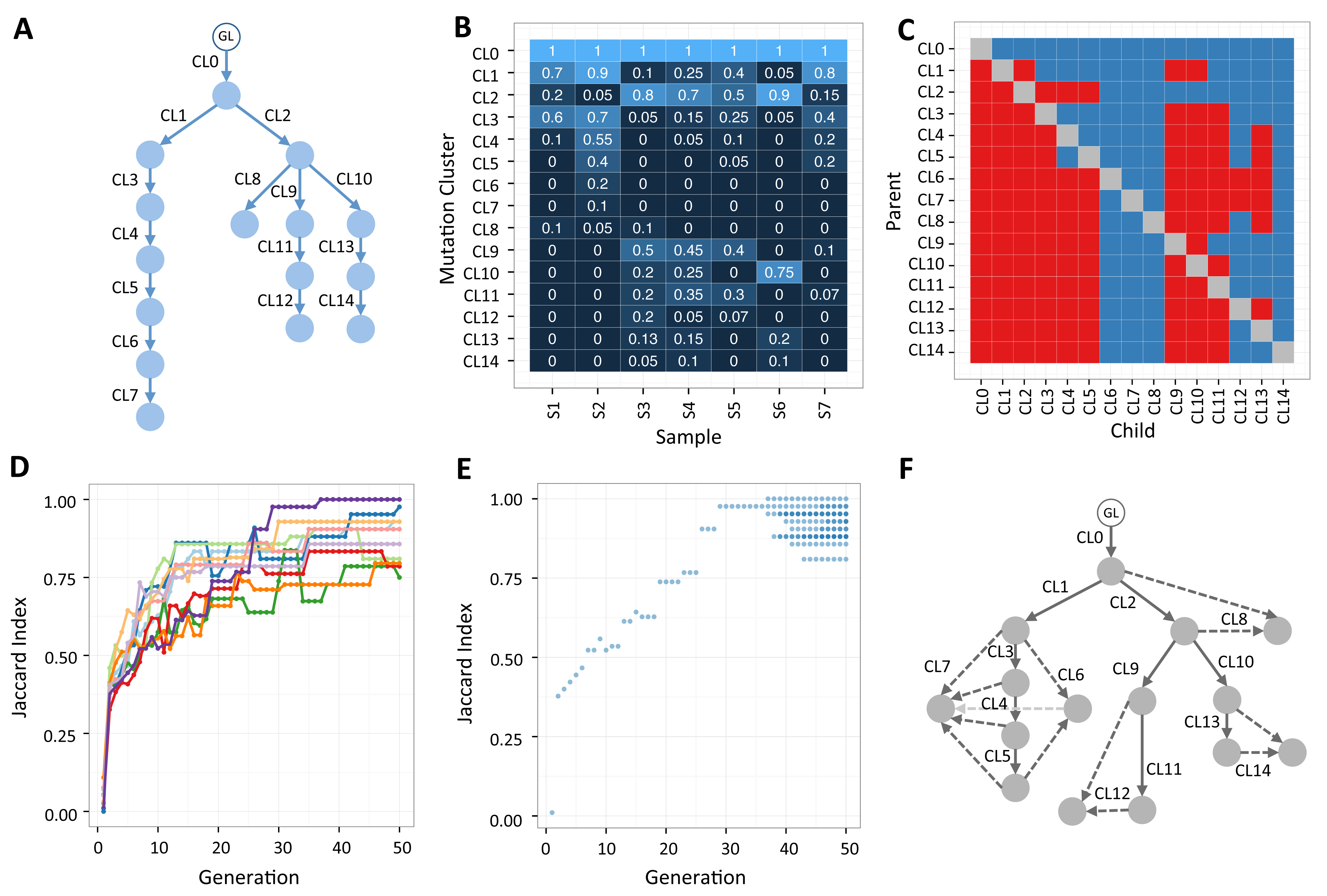

Supplement: S6 Fig — A. a phylogenetic tree summarizing clonal evolution in a second simulated patient B. Cellularity values of fifteen mutation clusters (CL0-14) in seven simulated biopsies S1–7 C. CPOV matrix depicting the hypothesis test outcomes. Each red square represents a pair of mutation clusters (I,J) for which the null hypothesis that I could be the parent of J was rejected. Each blue square represents a pair for which the null hypothesis could not be rejected. D. The Jaccard index (S1 Text, Topology Similarity Measure) of the true tree (A) and the maximum fitness tree(s) in population at the end of each GA generation. In cases where more than a single maximum fitness tree was present, the maximum of the Jaccard indices is plotted. Each color trace represent one of ten independent GA runs performed on inputs in (B, C). Only one of the ten GA runs (dark purple trace) identified the true tree (indicated by reaching Jaccard Index = 1). E. The Jaccard index of the true tree and the maximum fitness trees at the end of each generation for the successful run. Each transparent circle represents a single maximum fitness tree. This GA run also found 16 other phylogenetic trees sharing the maximum fitness with the true tree. (A). F. The consensus topology of the seventeen trees identified in (E). These trees disagreed in parental lineage of 5 out 15 mutation clusters CL16, CL7, CL8, CL12, CL14. (TIF) [file pcbi.1004416.s007.tif]

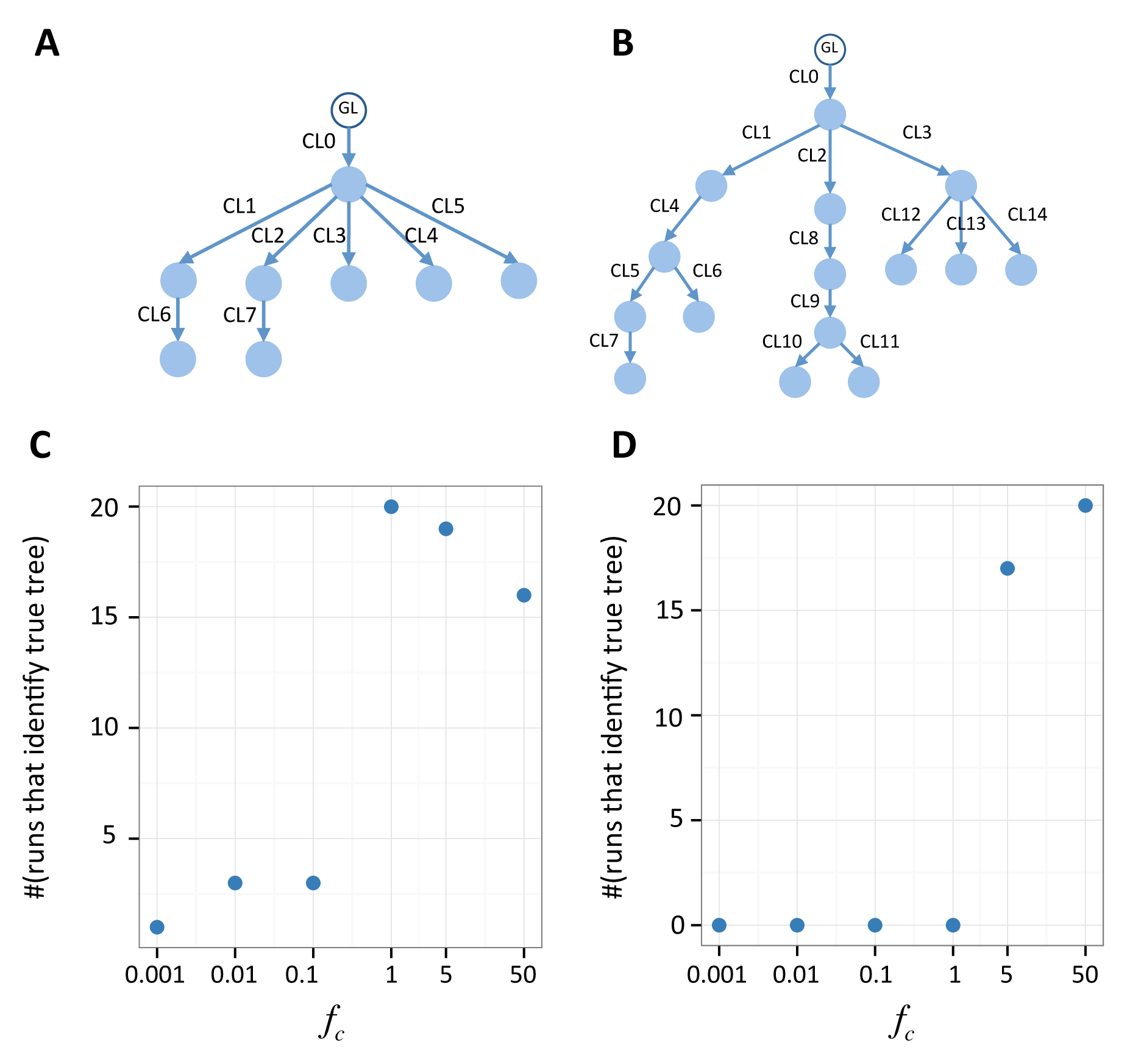

Supplement: S7 Fig — Simulations were designed to identify a reasonable default value of f c in a few scenarios. An 8-node tree and a 15-node tree were simulated (as described in the Simulations section of Methods). The GA was run 20 times for each tree. For the 8-node tree, each run spanned 20 generations and for the 15-node tree, each run spanned 50 generations to enable good sampling of the tree topology space. A 8-node tree B 15-node tree. C and D The number of runs (out of 20) in which the true tree was identified by the GA for six values of f c. The value f c = 5 produces good results for both scenarios. (TIF) [file pcbi.1004416.s008.tif]

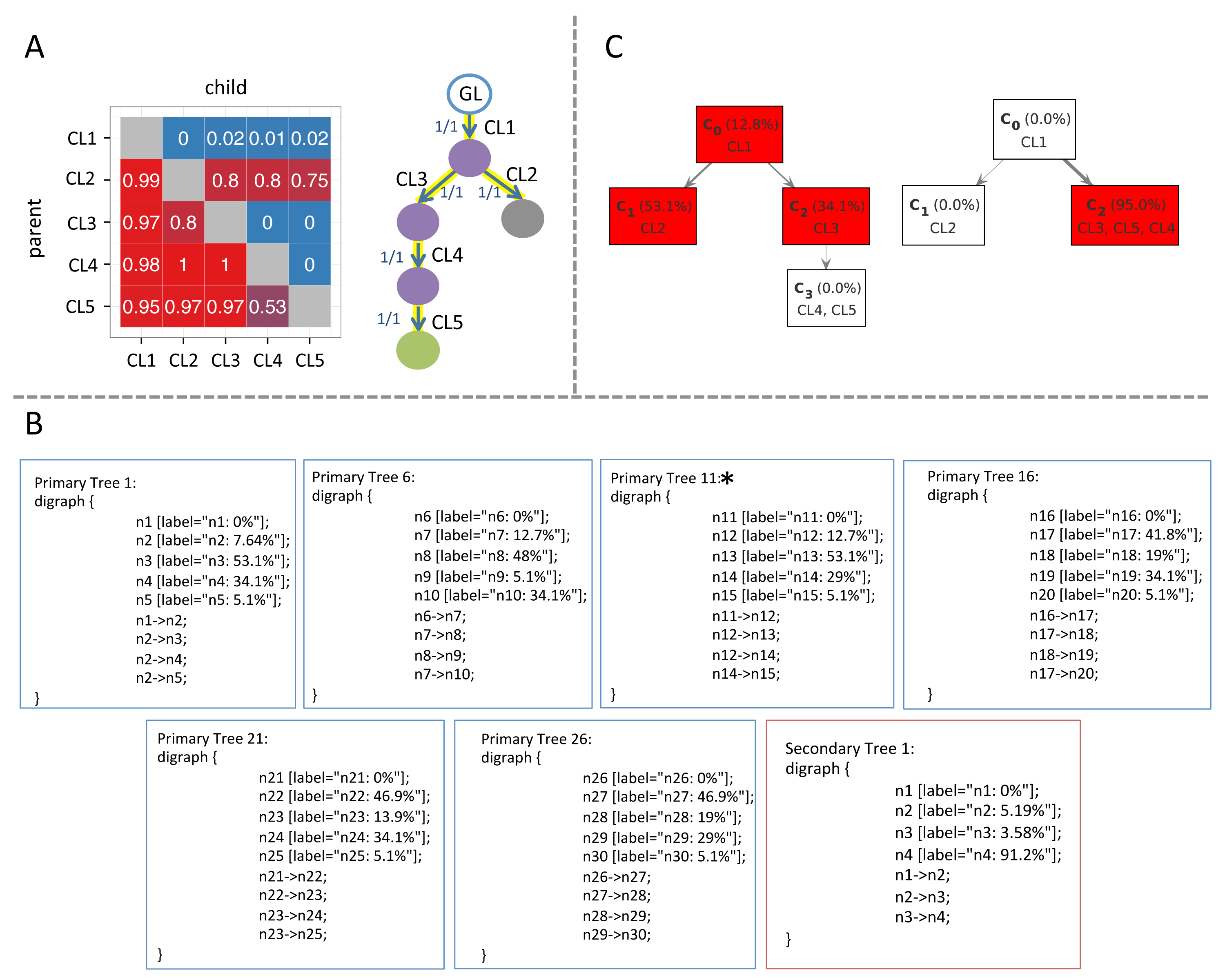

Supplement: S8 Fig — Subclone Seeker and TrAp have similar inputs to SCHISM but the modeling task and outputs are different. A SCHISM models a single, unified tree across multiple samples. B Subclone Seeker and C TrAp model trees for each individual sample. The AML1 patient has one primary and one relapse sample [12]. SubcloneSeeker reports six trees for the primary sample and one tree for the relapse (secondary) sample. Primary Tree 11 is reported as compatible with Secondary Tree 1 (marked with asterisks). TrAp reports a top-scoring pair of trees—one tree (left) for the primary and one tree (right) for the relapse sample. (TIF) [file pcbi.1004416.s009.tif]
