# Supplementary material for: SubClonal Hierarchy Inference from Somatic Mutations: Automatic Reconstruction of Cancer Evolutionary Trees from Multi-region Next Generation Sequencing
Source: PLoS Comput Biol. 2015 Oct 5;11(10):e1004416. doi: 10.1371/journal.pcbi.1004416 (PMC4593588; doi:10.1371/journal.pcbi.1004416)
Supplement: S1 Table — (PDF) [file pcbi.1004416.s010.pdf]

Table S1. Feature matrix comparing subclonal phylogeny reconstruction methods.

Method	Input Data	Decouples cellularity estimation and phylogeny reconstruction	Maximum number of tumor samples	Approach to handle multi-region samples	Goal	Search Strategy	Explicit Topology Bias	Output Data
SCHISM	mutation-to-cluster assignment, mutation / cluster cellularity, error estimate	Yes	no limit	inference of patient subclonal phylogeny tree by statistical integration of data across all samples	subclonal hierarchy inference	Genetic Algorithm	no built-in bias	cluster precedence order violation matrix, ranked set of unified all-sample tree topologies
Subclone Seeker [a]	mutation/ cluster cellularity	Yes	2	inference of sample subclonal phylogeny, identifying compatible sample trees	subclonal hierarchy inference	Exhaustive Enumeration	no built-in bias	unranked set of single-sample tree topologies, list of compatible pairs of single-sample trees, mutation co-localization frequency matrix
TrAp [b]	Mutation/ cluster cellularity, error estimate	Yes	no limit	inference of sample subclonal phylogeny with compatibility constraint across samples	subclonal hierarchy inference	Heuristic Search	prefers branched out topologies by favoring low number of populated subclones	ranked set of single-sample tree topologies
rec-BTP [c]	Mutation /cluster variant allele frequency, error estimates	Yes	1	NA	subclonal hierarchy inference	Heuristic Search	prefers branched out topologies by favoring low number of auxiliary nodes	unranked set of single-sample tree topologies
PhyloSub [d]	mutation read counts, copy number	No	no limit	inference of patient subclonal phylogeny tree by statistical integration of data across all samples	mutation cellularity inference and mutation to cluster assignment coupled with subclonal hierarchy inference	Bayesian Inference (MCMC)	no hard-coded bias	mutation to cluster assignment and cellularity estimates, ranked set of unified all-sample tree topologies, partial order plot

[a] http://genomebiology.com/2014/15/8/443
https://github.com/yiq/SubcloneSeeker
[b] http://nar.oxfordjournals.org/content/41/17/e165.long
http://sourceforge.net/projects/klugerlab/files/TrAp/
[c] http://bioinformatics.oxfordjournals.org/content/30/12/i78.abstract
http://compbio.cs.brown.edu/projects/btp/
[d] http://www.biomedcentral.com/1471-2105/15/35
https://github.com/morrislab/phylosub/
