# Supplementary material for: SubClonal Hierarchy Inference from Somatic Mutations: Automatic Reconstruction of Cancer Evolutionary Trees from Multi-region Next Generation Sequencing
Source: PLoS Comput Biol. 2015 Oct 5;11(10):e1004416. doi: 10.1371/journal.pcbi.1004416 (PMC4593588; doi:10.1371/journal.pcbi.1004416)
Supplement: S2 Table — (PDF) [file pcbi.1004416.s011.pdf]

Table S2. Detailed performance of cost function for multi-sample sequencing studies

	TC alone		MC alone		MC + TC	
Sample	#(maximum fitness trees)	includes manually crurated tree	#(maximum fitness trees)	includes manually crurated tree	#(maximum fitness trees)	includes manually crurated tree
AML1	6	Yes	1	Yes	1	Yes
AML15	2	Yes	2	Yes	2	Yes
AML27	1	Yes	1	Yes	1	Yes
AML28	6	Yes	1	Yes	1	Yes
AML31	2	Yes	1	Yes	1	Yes
AML35	1	Yes	1	Yes	1	Yes
AML40	1	Yes	1	Yes	1	Yes
AML43	2	Yes	1	Yes	1	Yes
CLL003	1	No	1	Yes	1	Yes
CLL006	24	Yes	2	Yes	2	Yes
CLL077	2	Yes	1	Yes	1	Yes
mSCLC 3588	762	No	1	Yes	1	Yes
mSCLC 3151	770	No	6	Yes	6	Yes
mSCLC 984	24	Yes	6	Yes	6	Yes
